# Novel experimental setup for megahertz X-ray diffraction in a diamond anvil cell at the High Energy Density (HED) instrument of the European X-ray Free-Electron Laser (EuXFEL)

**DOI:** 10.1107/S1600577521002551

**Published:** 2021-04-14

**Authors:** H. P. Liermann, Z. Konôpková, K. Appel, C. Prescher, A. Schropp, V. Cerantola, R. J. Husband, J. D. McHardy, M. I. McMahon, R. S. McWilliams, C. M. Pépin, J. Mainberger, M. Roeper, A. Berghäuser, H. Damker, P. Talkovski, M. Foese, N. Kujala, O. B. Ball, M. A. Baron, R. Briggs, M. Bykov, E. Bykova, J. Chantel, A. L. Coleman, H. Cynn, D. Dattelbaum, L. E. Dresselhaus-Marais, J. H. Eggert, L. Ehm, W. J. Evans, G. Fiquet, M. Frost, K. Glazyrin, A. F. Goncharov, H. Hwang, Zs. Jenei, J.-Y. Kim, F. Langenhorst, Y. Lee, M. Makita, H. Marquardt, E. E. McBride, S. Merkel, G. Morard, E. F. O’Bannon, C. Otzen, E. J. Pace, A. Pelka, J. S. Pigott, V. B. Prakapenka, R. Redmer, C. Sanchez-Valle, M. Schoelmerich, S. Speziale, G. Spiekermann, B. T. Sturtevant, S. Toleikis, N. Velisavljevic, M. Wilke, C.-S. Yoo, C. Baehtz, U. Zastrau, C. Strohm

**Affiliations:** a Photon Sciences, Deutsches Elektronen-Synchrotron (DESY), Notkestraße 85, Hamburg, Germany; b European X-Ray Free-Electron Laser Facility GmbH, Holzkoppel 4, 22869 Schenefeld, Germany; c School of Physics and Astronomy, Centre for Science at Extreme Conditions, and SUPA, University of Edinburgh, Peter Guthrie Tait Road, Edinburgh EH9 3FD, United Kingdom; d CEA, DAM, DIF, F-91297 Arpajon, France; e Université Paris-Saclay, CEA, Laboratoire Matière en Conditions Extrêmes, 91680 Bruyères-le-Châtel, France; f Helmholtz Zentrum Dresden Rossendorf e.V., 01328 Dresden, Germany; g Institut de Minéralogie, de Physique des Matériaux et de Cosmochimie (IMPMC), Sorbonne Université, UMR CNRS 7590, Musée National d’Histoire Naturelle, 4 Place Jussieu, Paris, France; h Lawrence Livermore National Laboratory, 7000 East Avenue, Livermore, CA 94550, USA; i Carnegie Science, Earth and Planets Laboratory, 5241 Broad Branch Road NW, Washington, DC 20015, USA; j Université de Lille, CNRS, INRAE, Centrale Lille, UMR 8207 – UMET – Unité Matériaux et Transformations, F-59000 Lille, France; k Los Alamos National Laboratory, Los Alamos, NM 87545, USA; l Mineral Physics Institute, Stony Brook University, Stony Brook, NY 11794, USA; m SLAC National Accelerator Laboratory, 2575 Sand Hill Road, Menlo Park, CA 94025, USA; n Department of Earth System Sciences, Yonsei University, 50 Yonsei-ro, Seodaemun-gu, Seoul 03722, Republic of Korea; o Department of Physics, Research Institute for High Pressure, Hanyang University, 222 Wangsimni-ro, Seoul 04763, Republic of Korea; p Institute of Geosciences, Friedrich Schiller University Jena, Carl-Zeiss-Promenade 10, 07745 Jena, Germany; q Department of Earth Sciences, University of Oxford, South Parks Road, Oxford OX1 3AN, United Kingdom; r Université Grenoble Alpes, Université Savoie Mont Blanc, CNRS, IRD, IFSTTAR, ISTerre, 38000 Grenoble, France; s Case Western Reserve University, 10900 Euclid Avenue, Cleveland, OH 44106, USA; t Center for Advanced Radiation Sources, University of Chicago, Chicago, IL 60637, USA; u Institut für Physik, Universität Rostock, D-18051 Rostock, Germany; v Institut für Mineralogie, University of Münster, Münster, Germany; w GFZ German Research Centre for Geosciences, Telegrafenberg, 14473 Potsdam, Germany; x Institut für Geowissenschaften, Universität Potsdam, Karl-Liebknecht-Straße 24-25, 14476 Potsdam, Germany; y Department of Chemistry, Institute of Shock Physics, and Materials Science and Engineering, Washington State University, Pullman, WA 99164, USA

**Keywords:** diamond anvil cells, X-ray free-electron lasers, high-precision X-ray diffraction, finite element modeling

## Abstract

The high-precision X-ray diffraction setup for work with diamond anvil cells (DACs) in interaction chamber 2 (IC2) of the High Energy Density instrument of the European X-ray Free-Electron Laser is described. This includes beamline optics, sample positioning and detector systems located in the multipurpose vacuum chamber. Concepts for pump–probe X-ray diffraction experiments in the DAC are described and their implementation demonstrated during the First User Community Assisted Commissioning experiment. X-ray heating and diffraction of Bi under pressure, obtained using 20 fs X-ray pulses at 17.8 keV and 2.2 MHz repetition, is illustrated through splitting of diffraction peaks, and interpreted employing finite element modeling of the sample chamber in the DAC.

## Abstract 2

The high-precision X-ray diffraction (XRD) setup for work with diamond anvil cells (DACs) in Interaction Chamber 2 of the High Energy Density (HED) instrument of the European X-ray Free-Electron Laser is described.

## 1. Introduction   

Generating high-pressure and high-temperature states of matter to better understand the dynamics of the interior of planetary bodies (Duffy *et al.*, 2015 ▸) such as the Earth (Mao & Hemley, 2007 ▸), or to synthesize new materials for industrial applications (*e.g.* Bykov *et al.*, 2018 ▸), has been an ongoing area of research for almost a century. One of the primary tools for creating these extreme conditions is the diamond anvil cell (DAC) which compresses a sample of interest between two opposing diamond anvils, while high temperatures may be induced through either heating internally, with infrared lasers (maximum 400 GPa and 5000 K), or externally, through the application of graphite or wire resistive heaters (maximum 200 GPa and 2000 K). The most powerful analytical tools to assess the crystallographic state of the sample at these extremes, and any changes it undergoes, have been X-ray powder and single-crystal diffraction performed at third-generation light sources. These provide a highly brilliant and tightly focused high-energy X-ray beam ideally suited to spatially resolving crystallographic changes in the samples. However, one of the major challenges encountered when studying reactive materials, particularly in heated DACs, is the possible reaction of the sample of interest with other materials in the sample cavity, such as the pressure-transmitting medium, surrounding gasket material or carbon released from the diamonds (Prakapenka *et al.*, 2003 ▸; Dewaele *et al.*, 2010 ▸; Morard *et al.*, 2018 ▸). These sample contaminations can result in significant discrepancies between data obtained in a DAC and those obtained using dynamic compression techniques such as gas guns and laser shock/ramp compression (*e.g.* Dewaele *et al.*, 2010 ▸; Morard *et al.*, 2018 ▸). Additional complications that may be encountered in static DAC compression experiments include gradual containment failure, sample movement, and fast recrystallization, all of which could be suppressed when performing the experiments faster.

In order to overcome these limitations when using a DAC, and to reach even higher pressures and temperatures, several research groups have conducted experiments using pulsed laser heating (Goncharov *et al.*, 2010 ▸; Aprilis *et al.*, 2017 ▸) and dynamically compressed DACs (Méndez *et al.*, 2021 ▸). While the development of time-resolved X-ray diffraction (XRD) detectors at third-generation sources is continuing (*e.g.* Hocine *et al.*, 2020 ▸) and one might soon be able to collect diffraction images at high energies at 24 kHz, and at even higher rates within a decade, the high-energy flux and brilliance offered at third- and future fourth-generation synchrotron sources will limit such studies to high-*Z* strongly scattering compounds such as Bi (Jenei *et al.*, 2019 ▸). In addition, significant work has also been vested in the development of elaborate sample assemblies to minimize unwanted reaction with pressure-transmitting media and to isolate the sample from the diamonds and reactive gasket materials, *e.g.* through Al_2_O_3_ disk insulators (*e.g.* Dewaele *et al.*, 2010 ▸; Ozawa *et al.*, 2016 ▸). These efforts have significantly improved our ability to collect contamination-free data on *e.g.* the melting temperatures of metals at high pressures, which ultimately allows for better comparison between static and dynamic compression experiments. However, the improved DAC experiments described above are still sparse and extremely challenging, in part because of the limited X-ray flux and time resolution available at third-generation light sources. For this reason, researchers in the high-pressure DAC community have been debating how to improve time-resolved X-ray experiments in the DAC.

Recent explorations of high-pressure states of matter using X-ray free-electron lasers (XFELs) have revolutionized our knowledge of materials’ structure and phase at extremes (*e.g.* Gorman *et al.*, 2015 ▸). To date, these femtosecond X-ray sources have been predominately used to probe dynamic compression experiments, where extreme pressures are produced by transient pressure waves, such as those generated by an optical laser pulse. However, with increasing demand for the wide range of dynamic measurements performed in a DAC, there is a realization that intense XFEL radiation will be extremely useful, and may be essential, to make headway in very fast dynamic DAC experiments. Current explorations of how best to integrate DAC techniques with XFEL sources focus on probing conditions of rapidly varying pressure and temperature states using piezoelectrically driven pressure cells (dynamic DAC or dDAC) and pulsed optical laser heating (*e.g.* Liermann, 2014 ▸; Liermann *et al.*, 2016 ▸), or the use of the X-ray source itself for dynamic excitation (Meza-Galvez *et al.*, 2020 ▸; Pace *et al.*, 2020 ▸).

This preliminary work suggests that an ideal instrument for such time-resolved studies is the High Energy Density (HED) instrument of the European XFEL (EuXFEL) in Schenefeld, Germany. The EuXFEL offers high-energy X-ray pulses up to 25 keV at a repetition rate of up to 4.5 MHz with a peak brilliance that is 10^8^ times higher than at any third-generation light source, with tight focusing to micrometre-scale beam spots. These properties are ideal for probing small samples through thick diamond anvils, with optimized access to *Q* space through limited apertures, *i.e.* for XRD.

Within this work we describe the experimental setup developed to conduct time-resolved XRD experiments with symmetric piston–cylinder DACs in interaction chamber 2 (IC2) of the HED instrument and its technical capabilities used during the First User Community Assisted Commissioning (1st UCAC; McWilliams, 2019 ▸). We also give examples of the first successful time-resolved diffraction experiments. We will conclude with an outlook on future developments and possibilities for high-pressure research using DACs at XFELs.

## 2. Concepts for time-resolved XRD experiments in a DAC at an XFEL   

**Figure 1:**
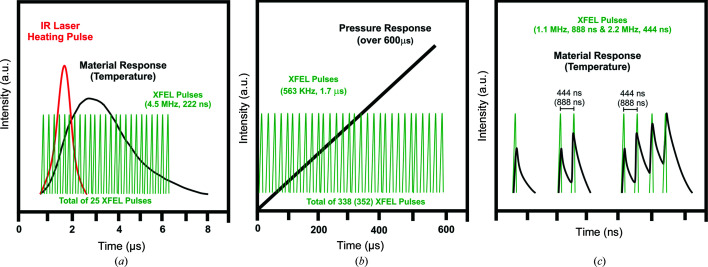
Schematic views of the expected timing for (*a*) infrared laser heated DAC and (*b*) dDAC experiments in the DAC setup for the HED instrument at the European XFEL. In the case of the dDAC experiment the limiting factor will be the fact that the AGIPD will only be able to collect 352 images. (*c*) Schematic of the single X-ray exposures and pump–probe approaches used during the 1st UCAC experiment. Consecutive diffraction patterns from one pulse train are accumulated in one image from the VAREX XRD 4343CT. The green pulses represent the X-ray pulses of 17.8 keV delivered within one train, while the black lines indicate the material response, such as an increase in temperature.

When designing time-resolved XRD experiments at an XFEL utilizing DACs, the timing structure of the XFEL beam and the detector capabilities both have to be considered. In the case of the EuXFEL, X-ray pulses are grouped in pulse trains at a repetition rate of 10 Hz, with each pulse train containing up to 2700 pulses, each pulse length ranging from 3–150 fs, and a maximum intra-train pulse repetition rate of 4.5 MHz (Fig. 1 ▸; Feng *et al.*, 2013 ▸). This means that any time-resolved XRD experiment can last up to 600 µs, the length of the pulse train. Because the accelerator of the EuXFEL serves three self-amplified spontaneous emission (SASE) sections simultaneously, the length of the pulse train is usually shorter for each instrument, and ranges from 200 to 600 µs depending on the demand from the different SASEs and the operational pattern of the accelerator.

An additional limiting factor is the number of diffraction images that can be collected on the XRD detector. In the case of the HED instrument, it has been proposed to use an adaptive gain integrating pixel detector (AGIPD; Allahgholi *et al.*, 2019 ▸) that can collect and store diffraction images at the EuXFEL repetition rate of 4.5 MHz, up to a maximum of 352 images per pulse train (10 Hz repetition rate). These images are then read out in the 99.4 ms time gap between pulse trains. At a 4.5 MHz repetition rate, the X-ray pulses are spaced 222 ns apart. However, the succession and the spacing between the pulses can be individually adapted to the needs of the experiment. For simplicity, at the beginning of operations, the repetition rate was tuned to a fraction of 4.5 MHz (*e.g.* 4.5, 2.25, 1.125, 0.75 and 0.563 MHz), increasing the spacing of the pulses to 444 ns, 888 ns, *etc*. The length of the pulse train from which data can be collected then depends on the number of diffraction patterns collectable on the AGIPD detector multiplied by the repetition rate, which should not exceed 200 (600) µs (Table 1 ▸).

These different pulse patterns can be used either to probe the sample response to an optical laser heating pulse, or during the fast compression of a sample in a dDAC; these examples represent the extreme cases for the timing of experiments proposed by Liermann *et al.* (2016 ▸). For example, in the case of optical pulsed laser excitation, a single pulse train can probe the initial state of a sample prior to arrival of the laser pulse, and the heating/cooling response of the sample, with a number of equally spaced X-ray pulses [Fig. 1 ▸(*a*)] at 4.5 MHz. In contrast, when conducting an experiment in a dDAC, it is possible to probe a ramped pressure increase with equally spaced pulses with a maximum length of 600 µs (338 pulses at 0.563 MHz) in the best case scenario, or less if higher repetition rates are required [Fig. 1 ▸(*b*)]. Based on initial simulations performed by Liermann *et al.* (2016 ▸), one can expect that each individual pulse containing up to 1 mJ of energy (equivalent to 3.5 × 10^11^ photons per pulse at 17.8 keV) is sufficient to generate a high-quality diffraction image, even in the case of low-*Z* compounds with a small scattering cross section.

Because one will be able to collect a diffraction pattern from each X-ray pulse, a set of full diffraction patterns will be obtained in the duration of a single infrared laser heating pulse of *e.g.* 5–25 µs, up to a maximum duration of 200 (600) µs employed for dDAC experiments. The possibility of performing single-shot/single-train experiments can potentially eliminate one of the major challenges in heated DAC work, *i.e.* the reaction of the sample with its surroundings, since contamination is a diffusion-driven process taking place on the millisecond or longer time scale, rather than on the microsecond scale.

The work by Liermann *et al.* (2016 ▸) also indicated that the sample can be heated by a single X-ray pulse to tens of thousands of kelvin via X-ray absorption. Thus, the attenuation of the X-ray beam has to be chosen carefully, especially for high-*Z* compounds, in order to avoid any significant heating that might compete with the heating delivered by the infrared heating laser. The calculations by Liermann *et al.* (2016 ▸) indicate that reduction of the fluence necessary to avoid heating is possible, without compromising the quality of the XRD images on the AGIPD detector. On the other hand, one might also take advantage of X-ray absorption to perform heating beyond what is a currently possible using a conventional infrared heating laser. This might be a very attractive alternative for heating a sample in a DAC, because the X-ray transparency of even optically opaque materials will enable X-ray absorption heating of the entire volume of the illuminated sample by the X-ray beam. Thus, in contrast to the optical infrared laser, which may only couple to absorbing (*i.e.* metallic) surfaces of the sample, bulk sample heating can be achieved without the need for heat to conduct through the sample, which could also have benefits in controlling and minimizing temperature gradients. X-ray heating in a DAC has been evaluated through finite element simulations in recent work by Meza-Galvez *et al.* (2020 ▸). This work proposes to utilize the different timescales of XRD and X-ray absorption, whereby XRD is immediate and occurs before any subsequent unit-cell expansion due to X-ray absorption. Thus, the following pattern emerges: the first X-ray pulse is used to collect a diffraction image of the unexcited state of the sample, before the structure heats up over the next tens of picoseconds via energy transfer from hot electrons. The next X-ray pulse would then probe the heated state of the sample 222 ns after first excitation (at 4.5 MHz), before heating the sample again, and so on. Using this stepwise heating approach, the sample could be heated to very high temperatures [*e.g.* Fig. 1 ▸(*c*)]. However, several competing effects will limit the heating of the sample to some finite temperature, such as (i) fast heat dissipation throughout the sample in the DAC chamber and (ii) significant heat loss due to the large thermal conductivity of the diamond anvils. Thus, the sample will cool in between the X-ray pulses. After a certain number of steps, the heating by X-ray absorption and cooling due to heat dissipation effectively reach the same magnitude and the sample cannot be heated to higher temperatures (Meza-Galvez *et al.*, 2020 ▸).

Due to the unavailability of the AGIPD detector at the time of the 1st UCAC (McWilliams *et al.*, 2019 ▸), we opted to collect diffraction images on two flat-panel detectors (VAREX XRD 4343CT) that cannot collect individual diffraction images from the pulses of a multi-pulse train, but instead collect diffraction images at 10 Hz, matching the repetition rate of the EuXFEL pulse train. Thus, XRD patterns generated by multiple X-ray pulses (*i.e.* up to 30 pulses at 1.1 and 2.2 MHz) within a pulse train are superimposed into a single diffraction image. While this detection scheme makes the identification of the individual diffraction images from each pulse within a pulse train more challenging, the present examples, where X-ray heating leads to clear and unambiguous shifts in line positions, serves as a proof of principle for serial diffraction measurements in a DAC. Furthermore, it enabled us to explore key questions, such as the stability of the diamond, sample and pressure medium, and effects such as X-ray induced fluorescence from the diamond and the sample, which complicate streak optical pyrometer (SOP) measurements for temperature estimation. We will give a short overview here of the initial findings from the 1st UCAC experiment, and a detailed description, evaluation and interpretation of the data collected will be presented in later publications.

## 3. Experimental setups in IA2   

**Figure 2:**
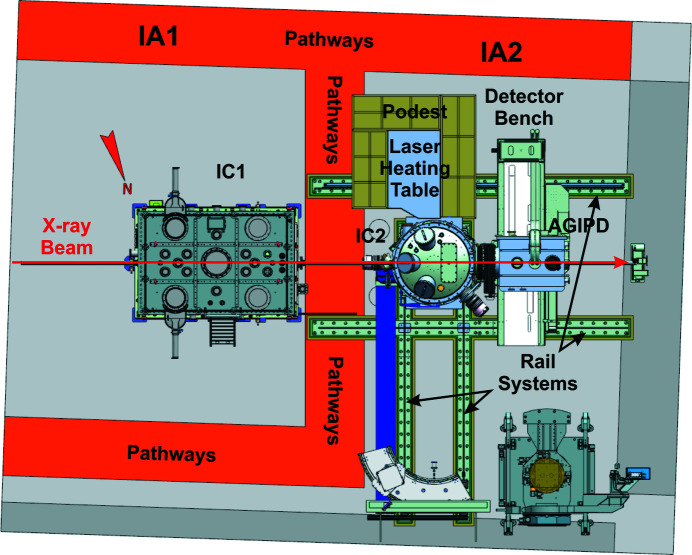
A 3D CAD illustration of the HED experimental hutch with IA1 (upstream) and IA2 (downstream). IA2 can host multiple sample environments, such as IC2 or the diffractometer for pulsed magnetic fields. Any of the portable sample environments can make use of a detector bench that provides space for the AGIPD for DAC experiments, as well as a multipurpose platform for smaller detector systems.

The HED experimental hutch at the EuXFEL provides two interaction areas (IAs), IA1 and IA2 (Fig. 2 ▸). IA1 houses the permanently installed interaction chamber 1 (IC1), while the multi-purpose IA2 provides space for portable sample environ­ments such as interaction chamber 2 (IC2) or *e.g.* a diffractometer for pulsed magnetic field studies. For the creation of excited states of matter, the HED instrument offers several drivers that can be operated in either or both of the IAs, such as the Amplitude short-pulse laser for the creation of relativistic plasmas exclusively within IC1, the DiPOLE (diode-pumped optical laser for experiments; Appel *et al.*, 2015 ▸; Nakatsutsumi *et al.*, 2017 ▸; Mason *et al.*, 2018 ▸; Banerjee *et al.*, 2020 ▸) long-pulse laser for the creation of cold and warm dense matter (CDM, WDM) in either IC1 or IC2, pulsed magnetic fields in IA2, and DACs in IC1 and IC2 for the generation of CDM and WDM. In all cases, the primary tool to probe the characteristics of the different excited states is the hard X-rays beam created by the EuXFEL. In the particular case of the DAC experiments that can be conducted in both IC1 and IC2, the emphasis of IC1 is on spectroscopic studies, whereas IC2 was designed for precision diffraction experiments with different large area-detector systems. Both IC1 and IC2 will also host setups for XRD for CDM and WDM created through ramp and shock compression via the DiPOLE or pump–probe (PP) lasers of the EuXFEL.

### 3.1. SASE2 and the X-ray energy spectrum   

X-rays for the HED instrument are provided by the self-amplified spontaneous emission section 2 (SASE2) of the EuXFEL, which is optimized for the generation of hard X-rays in the energy range 5–25 keV (Decking *et al.*, 2020 ▸). SASE2 is shared between the Materials Imaging and Dynamics (MID) and HED instruments, each operating 50% of the time.

**Figure 3:**
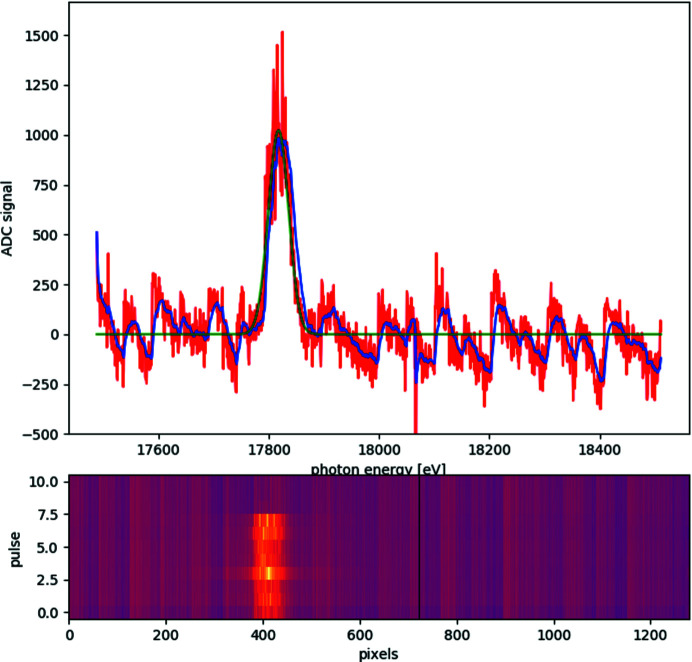
A typical SASE2 spectrum collected with an Si(111) crystal using the HIREX-II spectrometer at the beginning of the 1st UCAC experiment. The spectrum shows a central energy of 17.818 keV with a FWHM of 37 eV (± 4 eV) and a pulse-to-pulse jitter of 5 eV over eight consecutive pulses within a pulse train. The red line in the top graph shows data for a single pulse in a train, the blue line the smoothed data and the green line the corresponding Gaussian fit. The bottom graph shows the GOTTHARD detector image for a number of pulses in one train.

During the 1st UCAC experiment, the undulators were tuned to 17.8 keV, just below the zirconium *K* absorption edge at 17.998 keV, corresponding to an undulator gap of 16.2 mm. A typical SASE spectrum with a central wavelength of 17.818 keV, a bandwidth of 37 eV and a pulse-to-pulse jitter of 5 eV is depicted in Fig. 3 ▸.

The X-ray spectra were collected at the beginning of the 1st UCAC experiment on the high-resolution hard X-ray single-shot spectrometer II (HIREX-II spectrometer) which is installed in the XTD6 tunnel of SASE2 and provides spectral information for the HED instrument. HIREX-II is identical to the HIREX spectrometer installed at SASE1 (Kujala *et al.*, 2019 ▸, 2020 ▸), with the difference that it does not provide gratings which can be used as beam splitters. Thus, the spectrometer crystal has to be placed in the direct beam to collect energy spectra. For the spectral measurements an Si(111) crystal with (440) reflection as dispersive element and a GOTTHARD detector were employed. The spectra shown in Fig. 3 ▸ for eight consecutive pulses within a train demonstrate that the X-ray energy fluctuations from pulse to pulse within a train are of the order of Δ*E*/*E* = 2.9 × 10^−4^ with an energy spread of Δ*E*/*E* = 2 × 10^−3^, which agrees with the X-ray energy stability observed during the experiment (see Section 4.2). At the time of the 1st UCAC experiment the HIREX-II spectrometer was not available for more than ten pulses in one train for safety reasons. Therefore, the HIREX-II spectrometer was removed from the beam path for the actual experiments and hence no energy spectra were collected for the remainder of the beamtime. In future, the HIREX-II spectrometer will be available for pulse-to-pulse recording of energy spectra, which will be important to avoid any ambiguity related to the diffraction peak positions that directly influences the estimation of pressure and/or temperatures (see Section 4.3).

### 3.2. Focusing in IC2 and beam pointing stability   

**Figure 4:**
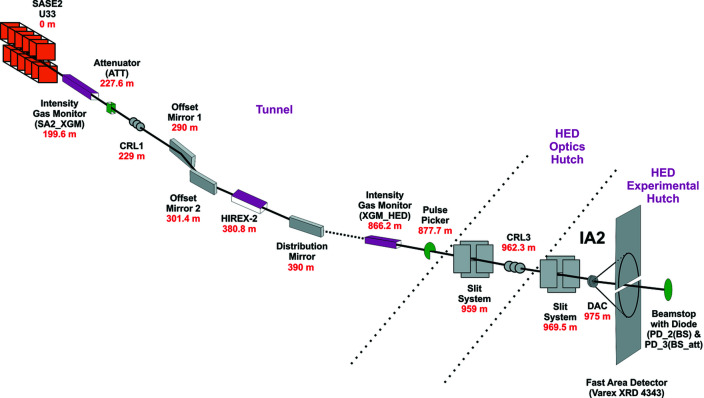
The optical train of the HED instrument used during the 1st UCAC experiment (proposal No. 2292).

The focusing concept at the HED instrument comprises three permanently installed sets of compound refractive lens (CRL) chambers which are equipped with ten cassettes of CRL holders that can each host up to ten lenses. Selection of lenses for the HED instrument was based on several parameters: optimal X-ray transmission, coverage of the energy range 5–25 keV, and the need for two different focal points in the HED experimental hutch, the target chamber center (TCC) in IC1 and in IC2. Two-dimensional X-ray lenses with bi-rotationally parabolic profiles made of Be of the grade IS50-M are used for focusing (Roth *et al.*, 2014 ▸, 2017 ▸). They have different radii ranging from 5.8 to 0.5 mm. In total, the beamline holds 115 lenses. The lenses are chromatic so that large radii enable collimation and focusing of low-energy X-rays. For hard X-rays, more lenses with smaller radii are used. The lens chambers are positioned at 229 m (CRL1), 857 m (CRL2) and 962.3 m (CRL3) from the undulator source (Fig. 4 ▸). Since the XFEL beam is coherent, the minimum beam size on the sample is diffraction limited. Direct focusing of CRL1 results in a minimum beam size of 260 to 160 µm FWHM at the TCC for energies from 5 to 25 keV, respectively (HED-TDR; Nakatsutsumi *et al.*, 2014 ▸). The lenses in CRL1 are generally used to collimate the beam or produce an intermediate focus for special focusing schemes. Its main purpose is to match the X-ray beam size to the aperture of the optical components of the beamline (*e.g.* mirrors and downstream CRL lenses). The focus of CRL2 offers a minimum spot size of 40–20 µm (FWHM) for 5–25 keV, respectively. The focal length varies from 5 to 25 m. Foci of 2.6 and 1 µm (FWHM) can be realized with CRL3 for energies of 5 and 25 keV, respectively, while the focal depth is around 20 mm at 5 keV and 63 mm at 25 keV. Focal points for IC1 and IC2 are located 9 and 12.7 m downstream from CRL3. In order to optimize the focal size at the TCC over the entire energy range (5–25 keV), CRL3 can be translated 490 mm along the beam. In addition to the permanently installed CRL1–3, a mobile CRL system can be installed in IC1 or IC2. This system offers a much smaller focal length so that diffraction-limited foci can be achieved with a size smaller than 500 nm.

**Figure 5:**
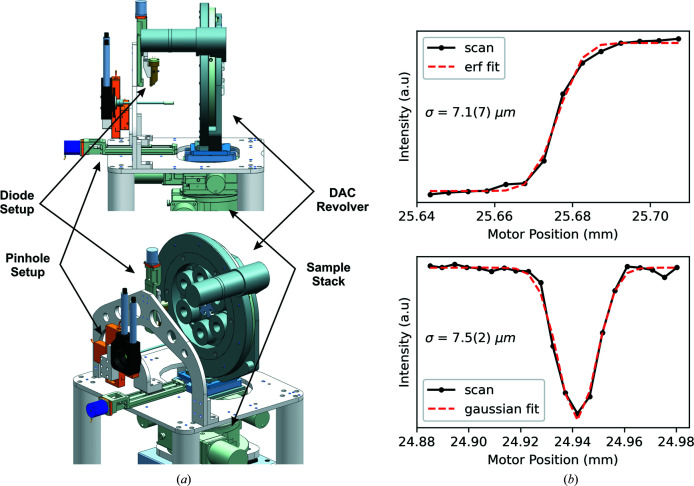
(*a*) A 3D model of the pinhole and diode *I*_0_ setup of the DAC setup in IC2. (*b*, top) Round-edge and (*b*, bottom) crosshair absorption scans collected by normalizing the intensity *I*_1_ of the DIM in the beamstop [PD_3(BS_att)] to the *I*_0_ of the DIM before the sample, using 14 keV X-rays during commissioning.

During the 1st UCAC experiment, the incident beam (17.8 keV) was collimated with CRL1 and focused with CRL3. The theoretical spot size for this combination of CRL1 and CRL3 is nominally 1–2 µm (FWHM in both horizontal and vertical) with a focal depth of 45 mm. The actual beam size determined during the experiment was 7.1 (7) µm in σ (16.7 µm FWHM) by scanning a polished steel round-edge tool through the focus of the X-ray beam (Fig. 5 ▸). This technique averages over several pulse trains and thus overestimates the focal spot size due to shot-to-shot fluctuations in the position of the focus. In future experiments better pointing stabilities should result in the expected theoretical focal size of 1–2 µm (FWHM).

A pulse picker is installed on the HED instrument to pick pulse trains at repetition rates of 10 Hz or lower. The pulse picker is a rotating blade of strongly absorbing material (sandwich of 2 mm B_4_C and 3 mm Densimed) with openings every 30°. It has been synchronized to the 10 Hz (or lower) repetition rates of the XFEL trains and may be used in shot-on-demand operation. The device is installed in the XTD6 tunnel at 877.7 m from the source and just downstream of the HED_XGM (Fig. 4 ▸).

### 3.3. IC2: optimized XRD at megahertz repetition rates in a DAC   

The IC2 vacuum chamber is designed to house the AGIPD detector for XRD at up to 4.5 MHz rate for samples compressed in a DAC, or two VAREX detectors at up to 10 Hz for XRD from laser shocked or ramp-compressed samples. The latter experimental setup will be discussed in future work after completion and commissioning of the DiPOLE high-energy laser. Within this section we describe the placement of the two VAREX area detectors for the collection of XRD patterns in the hertz regime from samples in DACs, as utilized during the 1st UCAC experiment in IC2 due to the unavailability of the AGIPD detector at the time of the experiment. Furthermore, we will describe plans for the installation of the AGIPD detector.

**Figure 6:**
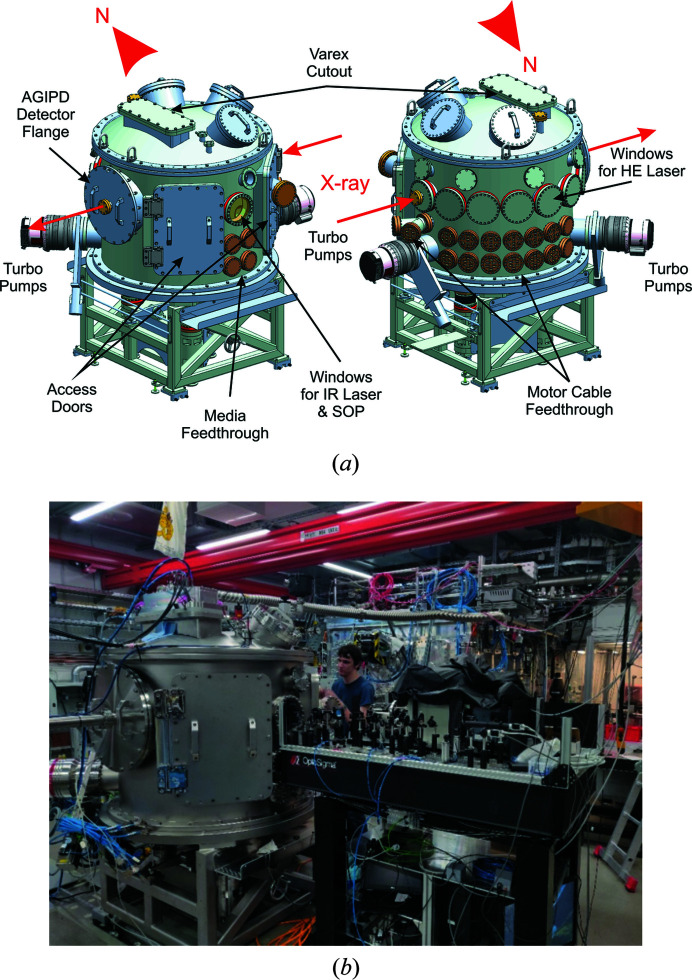
Interaction chamber 2 (IC2) in the HED instrument’s interaction area 2 (IA2). (*a*) Three-dimensional CAD drawings of the IC2 chamber without the supporting rail system, indicating the location of the different windows and feedthrough flanges. (*b*) A photograph of the IC2 experimental setup used for the 1st UCAC experiment.

IC2 has an outer diameter of 1360 mm and a height of 1520 mm (Fig. 6 ▸) and was manufactured by Pfeiffer Vacuum C&S. It consists of three parts: a bottom tub-type base, a middle cylinder and a top lid, all manufactured out of stainless steel and sealed with Viton O-rings. The modular design enables exchange of the three components for different applications, *e.g.* the lid can either accommodate the VAREX flat-panel detectors or a long working distance microscope. It also offers additional 300 mm CF-type ports for the attachment of additional analytical equipment in the future. The north side of the middle cylinder contains optical windows through which the DiPOLE laser will enter the chamber. West from the feedthroughs is a port for one of the two 1200 l turbo pumps (HiPace 1200 from Pfeiffer). The south side of the chamber contains two Viton-sealed rectangular access doors and an optical CF-type window flange. This flange serves as a window for viewing the sample optically from either side using dielectric turning mirrors (with precision-machined holes on the upstream and downstream sides for transmission of the beam), which also gives access for lasers and collection of optical emission from the sample induced by interaction with either X-rays or optical lasers. The optical system, located on a custom chamber-matching optical table [Fig. 6 ▸(*b*)], currently comprises optical microscopes, a pulsed or continuous infrared heating laser (model SP-100P-A_EP_Z, 1065 nm, from SPI Lasers), an SOP system (*e.g.* McWilliams *et al.*, 2015 ▸) using a Hamamatsu camera (model C13410 with s20 photocathode) coupled to a Princeton Instruments IsoPlane 160 spectrometer, integrated spatial filtering and sample illumination, with additional diagnostics for temperature measurements in preparation (Montgomery *et al.*, 2018 ▸). During the 1st UCAC experiments, the streak camera timing window ranged from 5 to 20 µs depending on the number of X-ray pulses in the XFEL train. Spectra of the optical light (in the 440–950 nm range) originating from the thermal and/or fluorescence light emitted from the interaction of the sample with X-rays were detected with time resolutions of the order of 10–100 ns, dependent on the streak window used, imaging configuration and signal. The optical system is calibrated with a standard tungsten incandescent lamp placed at the TCC, which allowed determination of the temperatures of hot samples by fitting the emission to a gray body Planck model. A detailed description of the observation system, including the SOP setup used to estimate temperatures, will be presented in more detail elsewhere.

The bottom tub base contains CF-type feedthroughs for the three legs of the experimental table that holds the sample stack for DAC alignment. The legs of the experimental table are mechanically decoupled from the vacuum chamber to prevent transfer of vibrations to the sample stack originating from the turbo pumps. In order to locate the experimental table reproducibly to the TCC of IC2, the legs are positioned on kinematic mounts recessed into the concrete floor of the HED hutch. The legs can be lifted off the kinematic mounts in order to move IC2. The tub base of IC2 is connected to a steel frame that sits on a rail system, which is also recessed into the concrete floor. This rail system enables the movement of IC2 towards the North side of the HED hutch, where the chamber may be ‘parked’. All three parts of the chamber offer a variety of KF-type ports used for connecting the roughing pump system of the HED instrument, vacuum gages to monitor the vacuum and valves to vent the chamber with dry nitro­gen.

The initial vacuum of the empty and baked-out IC2 obtained during its on-site acceptance test in January 2019 was 1.8 × 10^−7^ mbar (1 bar = 100 000 Pa) after pumping overnight. During the 1st UCAC experiment the vacuum reached a pressure of 5 × 10^−5^ mbar, as expected from a populated chamber (sample stack, DAC revolver, laser heating optics, clean-up slit) including DACs. The vacuum of the populated chamber is lower than the overall beamline vacuum. However, because the HED instrument has a differential pumping system up-stream from IC1, both IC1 and IC2 can be operated with vacuum as high as 10^−4^ mbar without jeopardizing the rest of the HED vacuum system. The turnaround time for venting the chamber, exchanging six DACs and reaching the above vacuum was as little as 20 min.

### 3.4. DAC alignment, observation system and pinhole setup   

**Figure 7:**
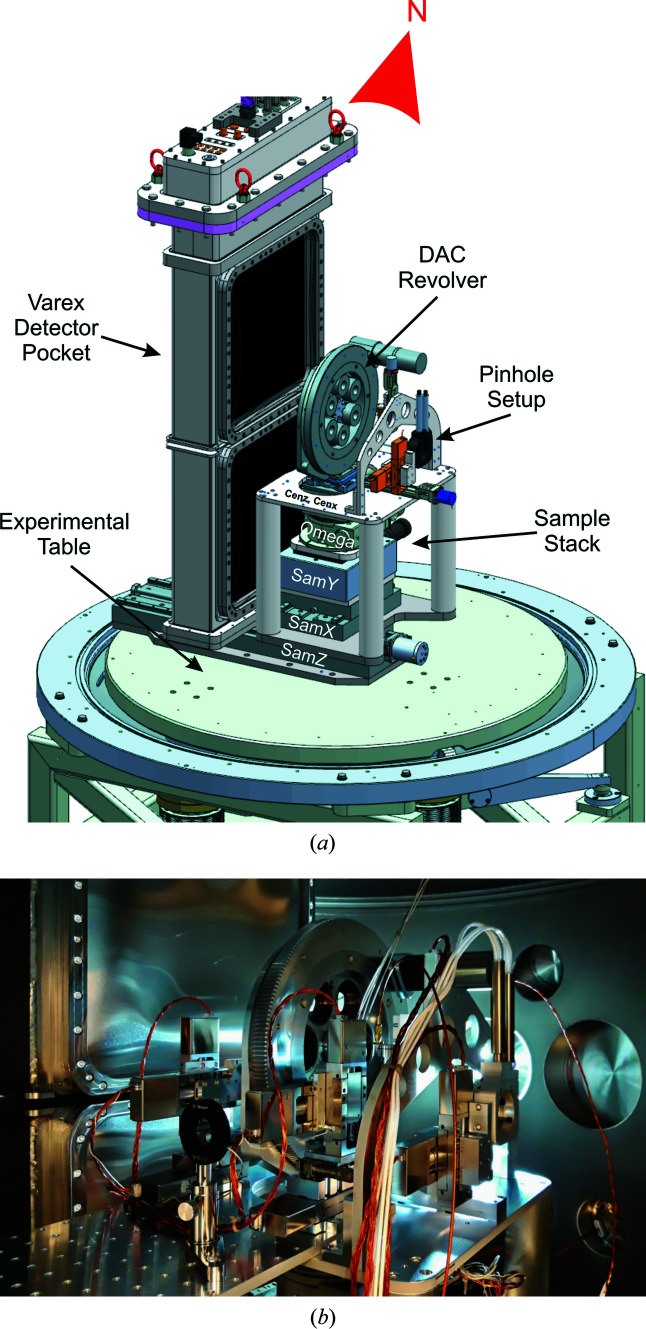
The sample stack and VAREX detector pocket inside IC2 used for the 1st UCAC experiment on the HED instrument. (*a*) A 3D CAD model of the inside of IC2 looking from the side. (*b*) A photograph of the inside of IC2, with the DAC setup while looking downstream.

In order to align DACs within IC2 reproducibly and with identical sample-to-detector distances (SDDs) for each DAC, a sample stack (Fig. 7 ▸), whose design is commonly used at high-pressure beamlines of third-generation light sources, was placed inside the vacuum chamber (*e.g.* Liermann *et al.*, 2015 ▸). With such a system the sample can be aligned onto the rotation axis by a simple triangulation method with the help of horizontal X-ray absorption profiles, ensuring an identical SDD of the DAC as that chosen for the calibration measurement. The translation at the bottom of the stack may be used to change the SDD and experimental configuration, *e.g.* to place the sample stack into the center of the chamber for usage with the VAREX flat-panel detector, or downstream close to the AGIPD detector. Details of the employed components are listed in Table 2 ▸. In order to reduce dead times for sample exchange caused by pumping down IC2, up to six symmetric piston–cylinder DACs may be placed on the sample stack at the same time through the use of a motorized revolver. The revolver holds membrane cups that place the membrane used for pressurizing on the downstream side of the symmetric DAC, enabling easy exchange of the DAC from the upstream side. While the revolver itself sits on a kinematic mount (BKL4, Newport) and may be exchanged in one piece, it is also possible to exchange the DACs individually when the revolver is located in IC2, avoiding potential misalignment of the observation system because of possible collisions. The latter option was used during the 1st UCAC experiment.

Due to strong horizontal beam jitter between trains during the 1st UCAC experiment it was not possible to use the triangulation method described above to align each sample to the rotation center. Instead, we centered the DAC through optical alignment of samples using the microscope also used to collect thermal emission, checking the beam position from damage observed on the gasket. Because of the refraction of the diamonds, a slight variance in microscope focal depth occurs with respect to the in-air XRD standard, and hence the SDD for each sample is adjusted using the known thickness of the anvils (determined prior to the experiment on beamline P02.2 at PETRA III, Hamburg, Germany).

One of the major challenges when performing high-pressure XRD experiments from samples in a DAC originates from the tails of the focused X-ray beam that create parasitic scattering from the high-*Z* gasket located between the diamonds surrounding the sample. For this reason, every dedicated high-pressure diffraction beamline offers an elaborate pinhole setup to eliminate the tails of the X-ray beam. Here we chose a pinhole setup similar to that used on beamline P02.2 at PETRA III (Liermann *et al.*, 2015 ▸). Because of the high intensity of the XFEL beam, standard pinholes used on third-generation sources (*e.g.* consisting of Pt) may not survive the extensive X-ray exposure. Thus, we developed a pinhole consisting of a sandwich of a 0.6 mm layer of B_4_C and a 0.4 mm layer of Ta with holes of varying diameters (0.04 to 0.02 mm). The latter are placed at the end of the 120 mm long tube that is fixed in the gimbal of the pinhole setup.

### 3.5. Intensity monitoring and scanning   

In order to perform absorption scans (*e.g.* Fig. 5 ▸), the intensity of the incident beam needs to be monitored before and after the sample. This is particularly important because of the natural intensity variations in the SASE process and the pointing instability of the X-ray beam. Thus, X-ray intensities need to be monitored close to the sample, after any clean-up systems or other optical elements of the beamline.

On the HED instrument, clean-up slits are located in the optics hutch and at the beginning of the experimental hutch at 16 and 5.5 m upstream from the TCC in IC2, respectively. The final clean-up pinhole to remove the tails of the focused X-ray beam at the sample position is located just before the sample (see Section 3.4). In order to monitor intensity fluctuations originating from the beamline optics, intensity monitors are located at several points of the approximately 1 km long beam transport in the SASE2 tunnels. Two absolute calibrated X-ray intensity gas monitors (XGMs) (Grünert *et al.*, 2019 ▸; Maltezopoulos *et al.*, 2019 ▸) are placed at the beginning of the SASE2 beamline (SA2_XGM) and inside the HED branch (HED_XGM). SA2_XGM measures the intensity of the X-ray pulses immediately after the undulators and HED_XGM gives the intensity values just before the beam enters the optics hutch. In addition, 2D monitors consisting of yttrium aluminium garnet (YAG) and diamond screens are positioned inside the HED tunnel, in the optics hutch and at several locations inside the experimental hutch. Scattering from the diamond screens is recorded on fast diodes (Hamamatsu S3590-09, diode intensity monitor or DIM) and can be used for X-ray beam intensity and position measurements. Downstream from the high-power clean-up slit system and CRL3 in the optics hutch, an *I*_0_ monitor consisting of a DIM is placed. The set of downstream monitors is completed with a DIM in the beam stop of the HED instrument at the end of the experimental hutch [PD_2(BS), PD_3(BS_att)]. The DIM in the beam stop can only record the X-ray beam intensity. Additional DIMs were placed before and after the pinhole setup during the 1st UCAC experiment. The DIMs are not absolute monitors and thus have to be calibrated at the respective photon energies using the calibrated XGMs without any optical elements in the beam path.

### 3.6. AGIPD and VAREX XRD 4343CT detectors   

IC2 is designed as a high-precision diffraction camera for DAC and laser shock-compression experiments. Thus, IC2 operates as a multi-purpose chamber to accommodate different experimental setups and two complementary detector systems: the HIBEF 1M adaptive gain integrating pixel detector (AGIPD) and two VAREX XRD 4343CT flat-panel detectors in a stacked configuration.

**Figure 8:**
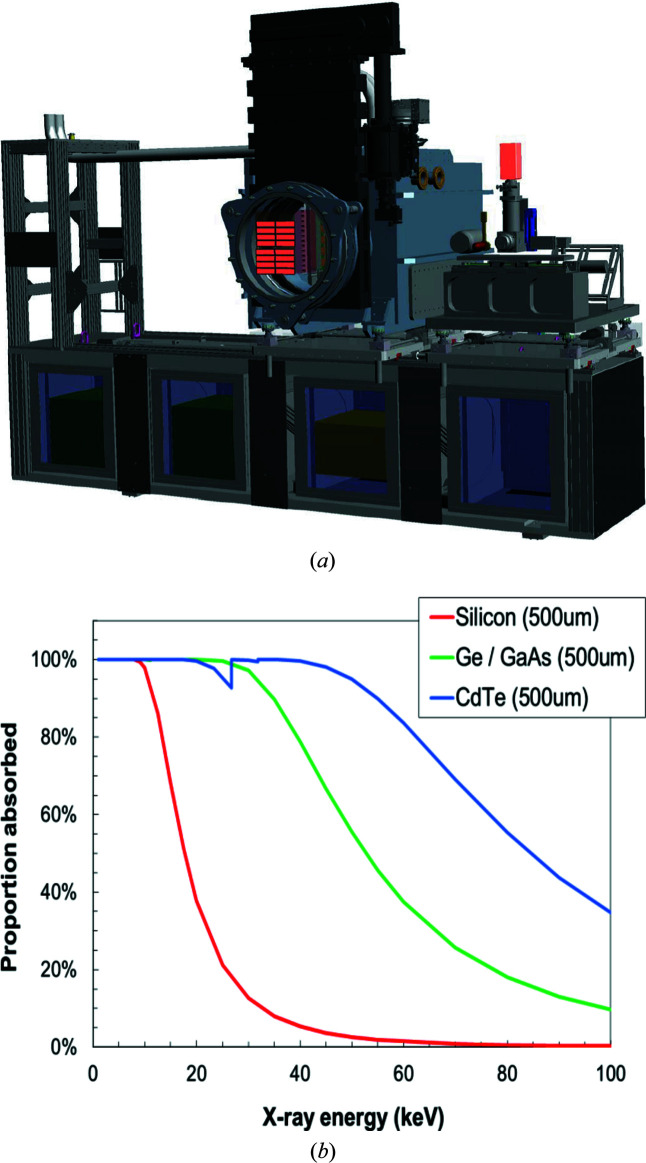
(*a*) A 3D CAD model of the detector bench in IA2 of the HED hutch. The detector bench offers motorized translations parallel and perpendicular to the X-ray beam in the horizontal direction by motorized rail systems that are located in the floor of the HED hutch and on top of the detector bench, respectively. The AGIPD detector may also be moved in the vertical direction through a system of four motorized jacks. Electronics racks below the detector bench platform house the external electronics of the above detectors, as well digitizers for the DIMs from IC2 and the beam stop. (*b*) Photoelectric absorption plots for different sensor materials as a function of X-ray beam energy. GaAs and CdTe sensor materials display an almost 100% absorption (quantum efficiency assuming a thickness of 0.5–1 mm) at 25 keV, which is the maximum energy that the EuXFEL may reach on the fundamental harmonic.

The AGIPD is capable of collecting XRD images with the maximum repetition rate of 4.5 MHz of the EuXFEL (Allahgholi *et al.*, 2019 ▸). For the DAC setup in IC2, a 1 Mpixel AGIPD is foreseen as the standard detector. Initially, the sensor material of the AGIPD will consist of silicon with 0.2 mm × 0.2 mm pixels, similar to the existing AGIPDs, while the final version will be equipped with a high-*Z* sensor material, such as GaAs, CdTe or CdZnTe, that offers higher photon absorption at higher energies in comparison with the silicon version [Fig. 8 ▸(*b*)]. In fact, at the maximum energy of 25 keV of the fundamental of SASE2, the photoelectron absorption of all high-*Z* sensor materials is almost 100%, assuming a thickness of 0.5–1 mm. The detector will be centered on the incident X-ray beam which passes through a central hole in the detector. Because the AGIPD may also be used for diffraction experiments in IC1 or IA2, it will be integrated on a mobile support structure, called the detector bench [Fig. 8 ▸(*a*)]. The support structure consists of motorized translations parallel to the X-ray beam recessed in the floor of the HED hutch and a translation on top of the detector bench, enabling movements of the detector perpendicular to the beam in the horizontal direction. Because the AGIPD encompasses in-vacuum electronics with permanent cooling, it requires an independent vacuum housing that can be attached to IC2 (or IC1) at a fixed position through the DN500 (see Section 3.3). The latter is connected through a 500 mm diameter bellow with a gate valve (HV-Shutter DN500, 19154-PE44-AMJ1, VAT). To avoid breaking the vacuum during sample exchange, the gate valve can be closed. Before closing the gate, the detector block of the AGIPD needs to be retracted into its housing via a motorized carrier system.

A second platform on top of the detector bench may house additional detectors such as the VAREX XRD 4343 detectors [not shown in Fig. 8 ▸(*a*)] or imaging cameras such as the PCO Edge (*e.g.* 4.2 CLHS) for phase-contrast imaging (PCI). Both platforms can move independently of each other.

**Figure 9:**
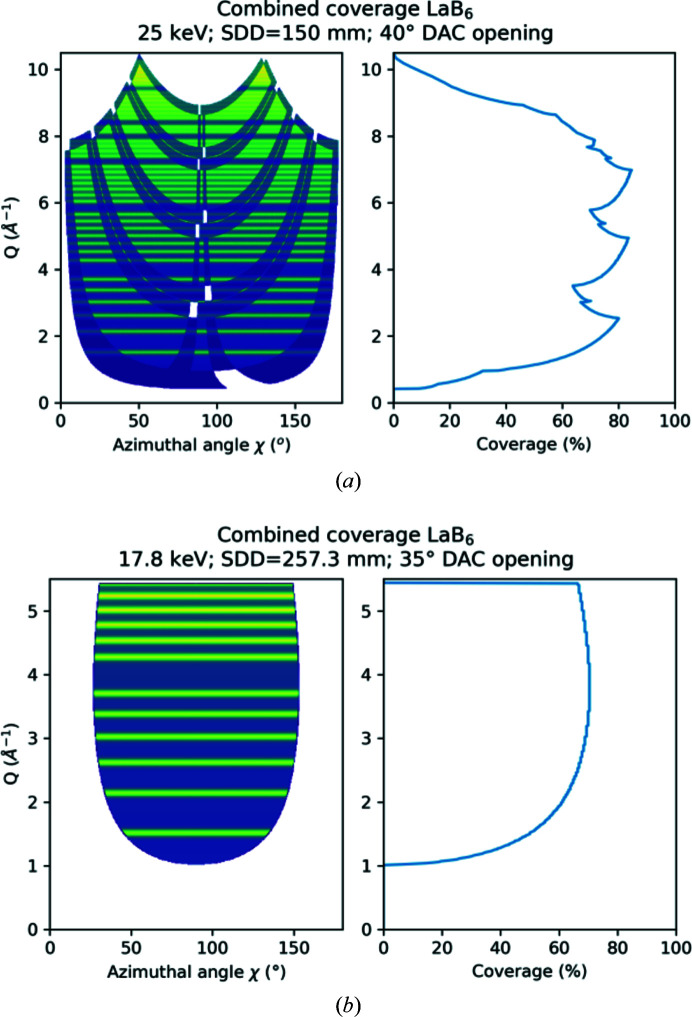
(*a*) Coverage of reciprocal space (in *Q*) at 25 keV on the AGIPD at an SDD of 150 mm and using a DAC opening angle of 35°. (*b*) Coverage of reciprocal space at 17.8 keV on the two VAREX XRD 4343CT flat-panel detectors at an SDD of 257.3 mm used during the first 1st UCAC experiment when using a DAC with a 35° opening angle. The lines in the unfolded diffraction patterns illustrate the positions of the LaB_6_ standard (660c, NIST) diffraction lines. To better illustrate reciprocal coverage with detector gaps, both sides (0–180° and 180–360°) in azimuthal angle have been projected on top of each other. The lighter blue/green areas in (*a*) are only covered on one side. Since the VAREX detector setup is symmetric, the upper and lower detectors provide equal coverage.

Liermann *et al.* (2016 ▸) suggested that the AGIPD should cover an angular range of ± 45° in 2θ to match the maximum opening of conventional DACs such as the symmetric piston–cylinder DAC (modified from LeToullec *et al.*, 1988 ▸) or the BX90 (Kantor *et al.*, 2012 ▸). Considering the active area of the AGIPD of ∼200 mm × 200 mm, the SDD needs to be approximately 150 mm to cover this angular range. This was achieved by displacing the DAC sample stack downstream from the TCC of IC2, as close as possible to the active area of the AGIPD. The resulting access to reciprocal space at the maximum X-ray energy of 25 keV provided by the principal harmonic of the HED instrument is depicted in Fig. 9 ▸ and listed in Table 3 ▸. Less access to reciprocal space, but a better instrumental resolution, may be achieved by increasing the SDD, either through moving the sample stack upstream or by retracting the detector downstream into its housing.

**Figure 10:**
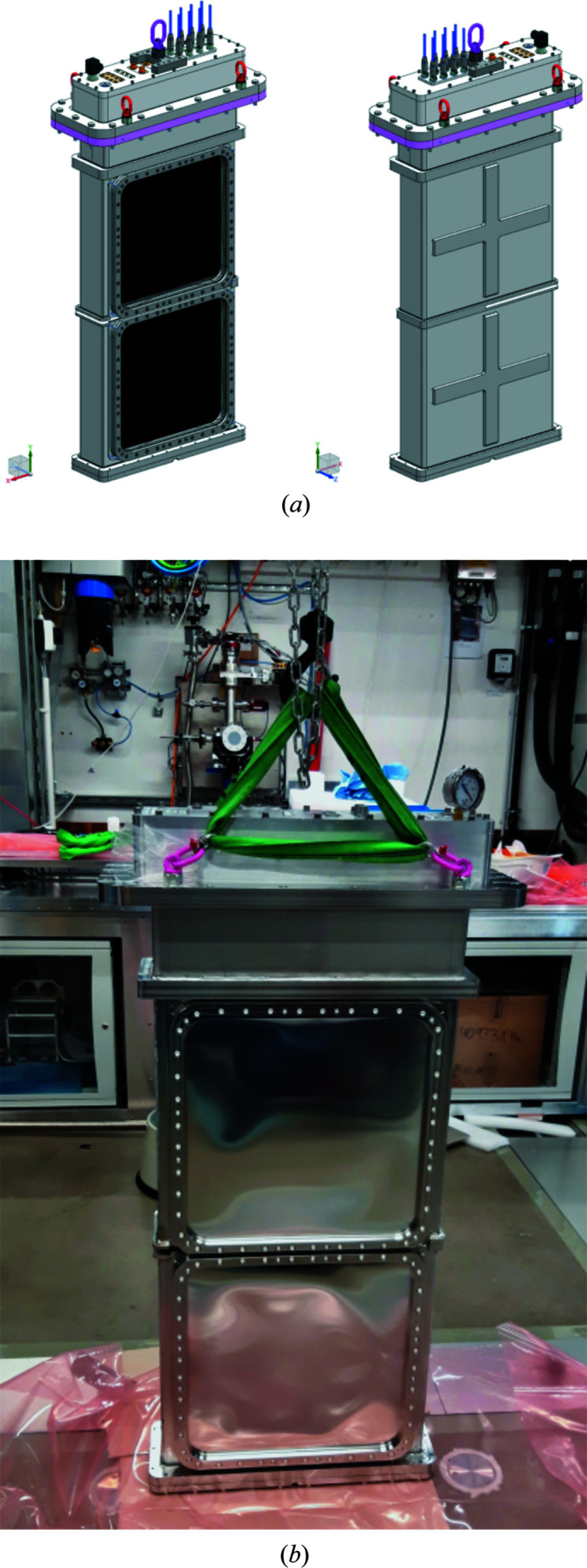
(*a*) Three-dimensional CAD models of the detector pocket for the VAREX XRD 4343CT flat-panel detectors and (*b*) a photograph of the actual setup outside IC2, including the Al window covering the detector panels.

**Figure 11:**
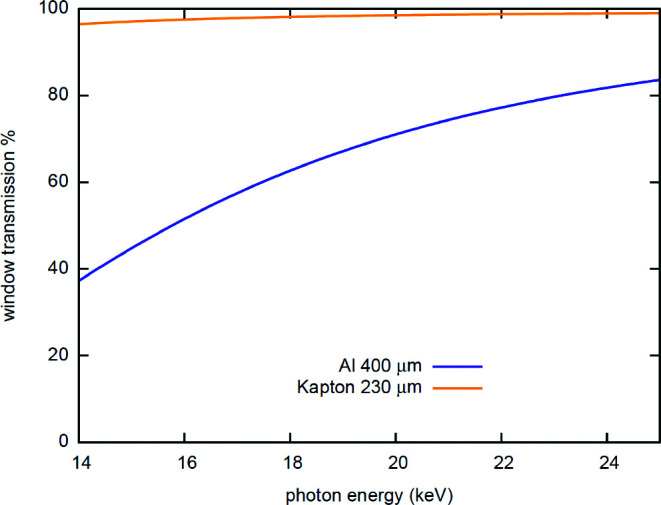
Absorption of the different window materials as a function of X-ray energy. While Kapton has a higher transmission it is not stable enough for operation in the vacuum chamber. For the 1st UCAC experiment a 0.4 mm Al window was successfully employed.

The second detector system available for XRD in IC2 consists of two VAREX XRD 4343CT flat-panel detectors that provide maximum gapless coverage and high quantum efficiency at high X-ray energies for both DAC and dynamic laser compression experiments at a pulse train repetition of 10 Hz. They consist of scintillator panels manufactured out of CsI:Tl oriented needle crystals which are bonded to a 2880 × 2880 pixel (pixel size 0.15 mm × 0.15 mm) thin-film technology diode array with an active surface of 432 mm × 432 mm. In order to provide 2θ coverage of 64.5° in the vertical, two detectors are placed one above the other inside IC2 at a distance of 220 mm from the TCC of IC2. Because these detectors are designed to operate in air, they are placed inside an air pocket equipped with thin metal or polyimide windows, inserted through the dedicated lid of IC2 (Fig. 10 ▸). After extensive testing of different window materials, aluminium (Al) with a thickness of 0.4 mm was selected as the most reliable, because of its limited deformation and high rupture stability during repetitive evacuation cycles. The X-ray absorption of the different window materials as a function of energy is shown in Fig. 11 ▸. The twin configuration of the two detectors with a horizontal gap in the equatorial plane was chosen in order to avoid parasitic scattering from the beam stop, to provide access to the direct beam for further analysis (*e.g.* PCI) downstream at the detector bench, and to permit intensity monitoring through the DIMs placed in the beam stop. The mid-plane separation for the active areas is 58 mm, resulting in a gap of 2θ = 7.9°. The flat-panel detector assembly can be rotated together with the lid around the center of IC2 in steps of 7.5°, defined by the hole pattern in the chamber top flange.

## 4. First user community assisted commissioning (1st UCAC) experiments and diffraction examples   

In October 2019 more than 40 researchers from 25 institutions, comprising a wide cross section of the static compression high-pressure community, came together under the umbrella of EuXFEL experiment No. 002292 (McWilliams, 2019 ▸) to perform the 1st UCAC experiment for DAC work at the HED instrument. A total of 70 DACs with different geometries, diamond anvils, samples and pressure-transmitting media (PTM) were prepared prior to the experiment. All samples were pre-characterized on beamline P02.2, the Extreme Conditions Beamline (ECB; Liermann *et al.*, 2015 ▸), at PETRA III, employing an 8 µm × 2 µm (horizontal × vertical) X-ray beam with an energy of 23.85 keV. Powder diffraction data were collected on a Perkin Elmer XRD 1621 flat-panel detector calibrated with a CeO_2_ standard (674b, NIST) through *Dioptas* (Prescher & Prakapenka, 2015 ▸) to ensure the quality of the sample and estimate the initial pressure. All data were recorded in DESY’s *Confluence* system to be available for the experiment at the HED instrument, as well as for post-experimental analysis. Only about half of the 70 DACs were examined in the XFEL beam, because several samples were prepared in a similar fashion with only slight variations (pressure medium and culet size) and many experiments were successful in the first instalment. While data analysis of the different samples by the individual research teams is ongoing, we will present here data collected on a sample of bismuth (Bi-I) in the DAC to demonstrate the data collection concept and the quality of the diffraction data obtainable.

### 4.1. Single and pump–probe diffraction images of Bi with an X-ray beam of 17.8 keV   

During the 1st UCAC experiment participants usually collected a single-pulse XRD pattern at low fluence to estimate the initial pressure of the sample, followed by two pulse diffraction patterns with a separation of either 444 ns (2.2 MHz) or 888 ns (1.1 MHz), as indicated in Fig. 1 ▸(*c*). In the two-pulse exposures, the first pulse probes the room-temperature sample and increases its temperature via X-ray absorption. The second X-ray pulse then probes the state of the sample 444 (888) ns after excitation from the first X-ray pulse. The VAREX XRD 4343CT detector collects the diffraction signals from both pulses in a single image. With increasing fluence (reduction in beamline attenuation) the second X-ray pulse probes the sample at higher and higher temperatures, and the combined diffraction image from the two pulses contains broadened and eventually split diffraction peaks. If the sample is entirely molten when probed by the second pulse, then the two-pulse diffraction pattern will comprise a room-temperature pattern and the diffuse scattering signal from the liquid. If the individual diffraction contribution of each exposure to the two-pulse image can be separated, the unit-cell parameters can be refined and the temperature of the heated sample estimated using a thermal equation of state (EoS). While many of our studies were made with a two-pulse pump–probe scheme, at the end of each study the sample was usually exposed to multiple X-ray pulses within a train in order to access higher temperatures.

As described earlier, the natural jitter of the SASE2 beam requires monitoring for the X-ray pulse intensity (energy) before and after the sample in order to characterize the beam, position the sample in the X-ray beam and correctly interpret the XRD data, as well as to determine the degree of heating of the sample. During initial commissioning of IC2 the calibrated X-ray intensity gas monitors (SA2_XGM, HED_XGM) and the diode signals at the pinhole and beam stop DIMs [PD_1(PH), PD_2(BS), PD_3(BS_att)] were available (Fig. 4 ▸) and used to perform normalized absorption scans to quantify the focal size and determine the crosshair position (Fig. 5 ▸). However, during the 1st UCAC experiment the pointing stability of the beam was insufficient for such scans, so the size of the beam was estimated through the fluorescence signal from a YAG crystal located at the sample position. These observations indicated some variability of the X-ray focus diameter in the range of 10–20 µm over the course of the 5 day experiment, and at times a pointing instability of roughly the FWHM diameter of the beam in the horizontal.

The thin diamond plate from the DIM in front of the sample was removed during the 1st UCAC experiment to provide maximum fluence to the different sample investigations. However, as pointed out in Section 3.5, it will be necessary in future studies to use the pinhole DIM [PD_1(PH)] to obtain an accurate reading of the X-ray intensity (energy) incident on the sample in order to estimate the temperature evolution within the sample as a result of each X-ray pulse. For this reason, the DIM signals need to be calibrated to represent the actual intensity (energy) of each X-ray pulse. The same will be true for the intensity-monitoring DIM in the beam stop [PD_2(BS), PD_3(BS_att)].

Despite the fact that the DIMs during the 1st UCAC experiment were not calibrated, one might still use the beam-stop DIM to assess the incident X-ray intensity on the sample. For this reason, diffraction patterns from a Ta foil were measured as a function of fluence between 5% and 100% and the intensity of the diffraction peaks plotted versus the beam-stop DIM [PD_2(BS)]. The resulting linear relationship confirmed that the beam-stop DIM [PD_2(BS)] gave a true estimate of the X-ray intensity (energy) incident on the sample. Furthermore, using the method of Liu (1982 ▸) it was possible to estimate the pulse energy from the ablation damage created in a freestanding 7 µm thick Ta foil as a function of fluence. By subsequently relating the calculated pulse energy to the value of beam-stop DIM PD_2(BS), and correcting for the X-ray absorption of the Ta foil, it was possible to estimate the pulse intensity (energy) for all subsequent exposures from the measured values of beam-stop DIM PD_2(BS), once it had been corrected for the X-ray absorption of each DAC examined during the 1st UCAC experiment, *i.e.* diamond anvil, sample and pressure-medium thicknesses.

The use of the X-ray intensity of beam-stop DIM PD_2(BS) also provided a method of estimating the jitter in the position of the focused beam from changes in the transmission factor of the Ta foil for a 20-pulse train of 100% fluence pulses. The first pulse is attenuated by the undamaged foil, reducing the value of beam-stop DIM PD_2(BS). This pulse also creates a hole in the foil as a result of ablation. The subsequent pulses (in the absence of jitter) should pass through this hole and result in larger values at beam-stop DIM PD_2(BS). While this was initially observed, subsequent pulses display a variety of values at beam-stop DIM PD_2(BS), varying from that expected from undamaged foil, to that expected from passing cleanly through a hole. This suggests that the jitter in the beam position corresponds to at least the FWHM of the beam itself, so that most pulses pass only partially though the hole, leading to further ablation of its edges and ultimately elongating the drilled hole in the Ta foil, especially in the horizontal. This observation will be important for the interpretation of all results of the 1st UCAC experiments. The entire procedure will be described in detail elsewhere (McHardy *et al.*, in preparation). In addition, the degree of damage induced in the Ta foils was used to estimate the size of the beam. Damage imprinting of single pulses in Ta foil implied a 13.3 (6) µm (FWHM) single pulse width, which agrees well with the round-edge absorption scan (see Section 3.2). Damage imprinting was also used to determine the position of the X-ray beam prior to sample exposure through alignment of the damage imprint on the gasket to the center of the optical microscope.

Finally, because the position jitter of the X-ray beam was more than the FWHM of the incident X-ray beam, the pinhole setup was not utilized during the experiment. This was acceptable for many DACs used in the 1st UCAC experiment because they were equipped with large diamond culets (0.8–0.2 mm). Thus, the few DACs containing samples compressed to 1 Mbar or above did show significant scattering from the gasket (rhenium, tantalum or stainless steel) in the diffraction images. With improved pointing stability, future diffraction experiments will use clean-up pinholes to reduce the parasitic scattering of the gasket materials.

### 4.2. Instrumental resolution of the VAREX XRD 4343CT detectors   

For the 1st UCAC experiment the VAREX XRD 4343CT flat-panel detectors were placed at an SDD of 257.3 mm when the sample stack was located 8 mm upstream from the TCC of IC2. The resulting angular coverage and access to reciprocal space at the X-ray energy of 17.8 keV during the 1st UCAC experiment and a maximum opening of 2θ = 45° and the actual opening of 35° of the DAC are depicted in Fig. 9 ▸ and Table 3 ▸.

**Figure 12:**
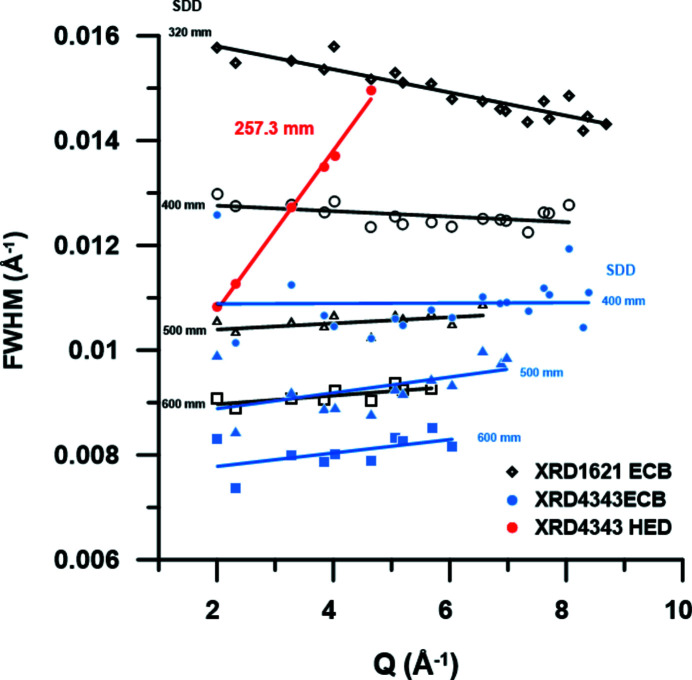
Comparison of the FWHM of the diffraction peaks of the CeO_2_ standard (NIST) collected on beamline P02.2 at PETRA III and the HED instrument at the EuXFEL. Comparison of the IR collected on P02.2 at 25.6 keV with a Perkin Elmer XRD1621 (open black symbols) and a VAREX XRD 4343CT (solid blue symbols), and that of the HED instrument at 17.8 keV collected on the VAREX XRD 4343CT (red solid circles) based on the summation of 100 individual patterns. Solid black, blue and red lines are linear fits to the data to guide the eye.

During the 1st UCAC experiment, a CeO_2_ standard (674b, NIST) was used to calibrate the SDD and orientation of the two VAREX XRD 4343CT flat-panel detectors. Using these diffraction patterns one can compare the instrumental resolution (IR) with those of standard high-pressure diffraction beamlines, such as P02.2 at PETRA III that also uses CRLs for focusing and similar flat-panel detectors, *i.e.* the Perkin Elmer XRD 1621. Fig. 12 ▸ shows that in general the VAREX XRD 4343CT flat-panel detector has a slightly better IR than its predecessor, the Perkin Elmer XRD 1621. The plot compares the FWHM of the peaks from the diffraction pattern of the CeO_2_ standard collected at 25.6 keV with a CRL-focused beam on the ECB for both detectors and at different SDDs. Based on the discussion by Liermann *et al.* (2015 ▸) and Jenei *et al.* (2019 ▸), the improved IR is due to the decreased pixel size of the XRD 4343CT. Fig. 12 ▸ also shows the IR as derived from the CeO_2_ pattern collected at 17.8 keV with a CRL-focused beam on the HED instrument. The IRs are very different: while that on the ECB is almost flat and even negative at very small SDD (pixel size controlled), the IR from the HED instrument shows a steep increase as a function of scattering vector *Q*. Since the divergence of the HED beam (45 µrad based on theoretical calculations) due to focusing is relatively small compared with the ECB [12.8 m (HED) from the sample point versus 1.2 m (ECB)] it cannot be the controlling factor of the IR at the HED. Furthermore, it is unlikely that the pixel size of the VAREX controls the IR since it is smaller for the HED detectors, as discussed above (Fig. 12 ▸). The most likely origin of the broadening of the Bragg reflections with increasing *Q* is the pink-beam character of SASE2 (Fig. 3 ▸). By simulating the diffraction patterns of the CeO_2_ standard, assuming a constant intrinsic FWHM of the Bragg reflections with *Q*, a Gaussian energy distribution with a FWHM of 40 eV centered around 17.8 keV and identical SDDs, it was possible to reproduce the steep increase in the IR as function of increasing *Q* after integrating the sum of the different XRD peaks.

It may be pointed out that the IR for the AGIPD detector is likely to be even less favorable compared with the VAREX detector because of the increased pixel size of the AGIPD detector (0.15 mm × 0.15 mm for the VAREX compared with 0.2 mm × 0.2 mm for the AGIPD). However, this can be compensated by increasing the SDD through retracting the detector module downstream or by moving the sample stack further upstream, at the expense of accessible *Q* range.

The jitter in the X-ray energy for each pulse can be determined from the changes in the peak positions in the CeO_2_ diffraction patterns, and is of the order of Δ*E*/*E* = 2 × 10^−4^, which is in good agreement with the in-train jitter estimated from the energy spectrum of Δ*E*/*E* = 3 × 10^−4^ (see Section 3.1). However, the X-ray energy jitter from pulse train to pulse train can vary more widely, as we will demonstrate in Section 4.3 when looking at the XRD data from Bi-I.

### 4.3. Diffraction from Bi-I   

**Figure 13:**
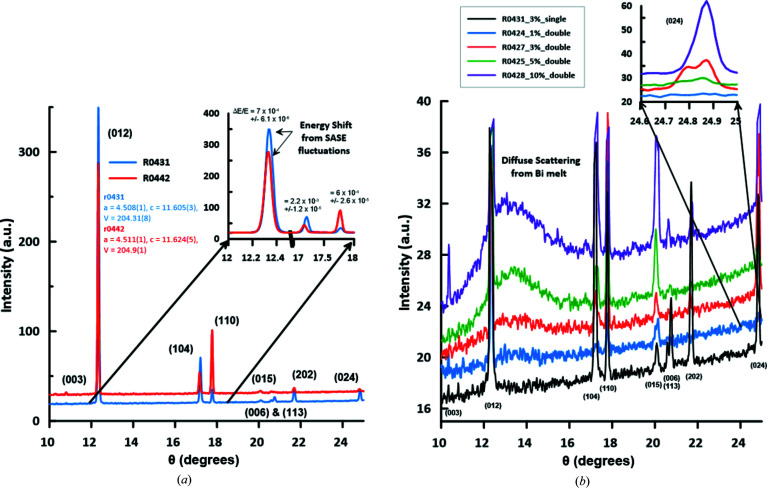
Integrated diffraction patterns of Bi-I collected on the VAREX XRD 4343CT, demonstrating the high quality of the diffraction patterns obtained from a single X-ray pulse. The diffraction patterns are not background subtracted and are stacked for better visibility. (*a*) Diffraction patterns of Bi-I collected using a single 20 fs (r.m.s. as determined at 0.25 nC) X-ray pulse at 17.8 keV and at 3% transmission (4 µJ per pulse) where Bi was compressed to 1.49 (2) GPa in a symmetric diamond anvil cell. Variations in the positions of the diffraction peaks are caused by the energy fluctuations of the SASE energy spectrum. Intensity fluctuations in the peaks are due to crystallite orientations changing as a consequence of sample melting and recrystallization. (*b*) Diffraction patterns of Bi-I collected with two sequential X-ray pulses (r.m.s. as determined at 0.25 nC), 444 ns apart with varying transmission settings. For comparison a single-pulse pattern is also shown, which indicates that even at the lowest transmission of 1% part of the sample had melted following the first pulse, as evident from the diffuse scattering.

A 10–15 µm strip of Bi-I was extracted from a 1 mm thick foil (99.999% purity Alfa-Aesar, stock No. 41636) and loaded with 10–15 µm LiF platelets as an X-ray transparent PTM in a symmetric piston–cylinder type DAC with a sample chamber of 0.03 mm thickness (compressed to 0.028 mm during loading) and a diameter of 0.13 mm. The thickness of the sample and thus the thickness of the pressure medium was confirmed by X-ray absorption scans after the experiment, with the Bi foil having an average thickness of 14–15 µm. The upstream side of the DAC was equipped with a type Ia standard design diamond and the downstream side with a type IIa Boehler Almax cut diamond, both with a culet diameter of 0.3 mm. The initial pressure of the sample was estimated to be 1.7 (2) GPa, based on the XRD pattern collected during screening on the ECB and employing the EoS for Bi-I derived by Degtyareva *et al.* (2004 ▸). During transport and installation of the DAC at the EuXFEL 12 days later, the pressure had dropped to 1.49 (1) GPa, as determined from the XRD pattern collected at the HED instrument based on the same EoS for Bi-I. Fig. 13 ▸(*a*) shows two diffraction images of Bi-I at 1.49 (1) GPa collected with a single 20 fs (r.m.s. as determined for 0.25 nC electron bunch charge) 17.8 keV X-ray pulse at 3% transmission (∼4 µJ per pulse). While both patterns were collected at almost identical pressures, the diffraction peaks in pattern R0442 are shifted to lower 2θ values. This can be interpreted as a drop in pressure to 1.38 (2) GPa, an increase in the sample temperature, or a result of the energy jitter of the SASE beam (Table 4 ▸). For a cubic material, one would be unable to decide between these three possibilities. However, Bi-I is rhombohedral and one can use the *c*/*a* ratio, which is strongly pressure dependent (Degtyareva *et al.*, 2004 ▸) but not X-ray energy dependent, to estimate the sample pressure. Using this approach, the pressure estimation is independent of variations in the X-ray wavelength. The *c*/*a* ratio obtained using the two diffraction patterns was identical within errors, indicating that the pressure of the Bi-I sample was unchanged. Thus, the shift in peak position is not related to a decrease in pressure (or increase in temperature) but has its origin in the energy jitter of the SASE beam, which for Δ*E*/*E* ≃ 1 × 10^−3^ estimated from the diffraction peak positions equates to 18 eV.

Fig. 13 ▸(*b*) illustrates the diffraction patterns collected from two-pulse X-ray exposures at 2.2 MHz. At a transmission of 1%, single peaks are observed, indicating a lack of detectable residual heating after 444 ns (the arrival time of the second pulse). However, at 3% transmission the splitting of the diffraction peaks can be resolved (especially at higher 2θ), indicating residual heating of Bi-I as probed by the second X-ray pulse. In addition, first signs of diffuse scattering from liquid Bi can be observed. At 5% and 10% transmission, strong diffuse scattering and disappearance of peak splitting at 10% transmission, with line positions matching those at lower power, indicate that most of the exposed sample was molten at the time of arrival of the second X-ray pulse. Fitting this sequence of XRD patterns shows that the first pulse probed cold Bi-I and the second the increasingly heated sample; the unshifted peaks indicate a pressure of 1.7 (1) GPa at 300 K, indicating some annealing after the reference single-pulse shots.

We used the thermal expansion measured between 5 and 516 K at ambient pressure (Fischer *et al.*, 1978 ▸) to estimate the temperature of hot Bi-I probed during the second X-ray pulse at 1.7 (1) GPa. This assumes that the pressure dependence of the thermal expansion is negligible between ambient pressure and 1.7 (1) GPa. Using this very simple approximation, temperatures of 552 (6) K at 1.68 (1) GPa and 551 (2) K at 1.619 (1) GPa were estimated for 3% and 5% transmission, respectively. These temperatures are somewhat higher than the melting temperature of 470 (10) K at 1.4–1.7 GPa reported in the literature (*e.g.* Lin *et al.*, 2017 ▸ or Ono, 2018 ▸). This is not likely to be a manifestation of melting kinetics (Gorman *et al.*, 2015 ▸), due to the long pump–probe delay, but may be related to the extrapolations made in the EoS, ambiguities in the refinement of hot and cold diffraction patterns superimposed in one diffraction image, or pressure reductions at high temperatures resulting from the density increase upon re-entrant melting (leading to both an increased melting temperature and a reduced volume of the residual solid). Pattern collection at megahertz rates using the AGIPD will provide individual pump and probe diffraction images for the cold and heated states to resolve this ambiguity better. Thus, in the future one might be able to use this approach together with a high-quality thermal EoS of an internal standard to estimate the thermal expansion and melting temperatures of unknown samples.

### 4.4. Energy on the target and sample temperature   

Total pulse energies on the target (*E*_target_) are estimated for each pulse from the amplitude (in analog-to-digital units, ADUs) detected by beamstop diode PD_2 or equivalently the secondary diode PD_3 [multiplied by the sensitivity factor 7.743 (5)], as needed when PD_2 is saturated. The diode readings were calibrated to ablation damage imprints in freestanding Ta foil (McHardy *et al.*, in preparation) and follow the relationship
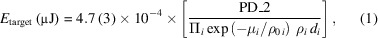
where the product of the attenuations of all target layers appears in the denominator: *i* indicates a layer, μ is its attenuation coefficient at standard density ρ_0_, ρ is the actual density (accounting for any pre-compression) and *d* is its thickness. These calculations assume that the layer is not disrupted by preceding exposures, a good assumption for samples compressed in the DAC. The transmission on the target downstream from SA2_XGM and HED_XGM without additional filtration is estimated to be 20% and 30%, respectively.

**Figure 14:**
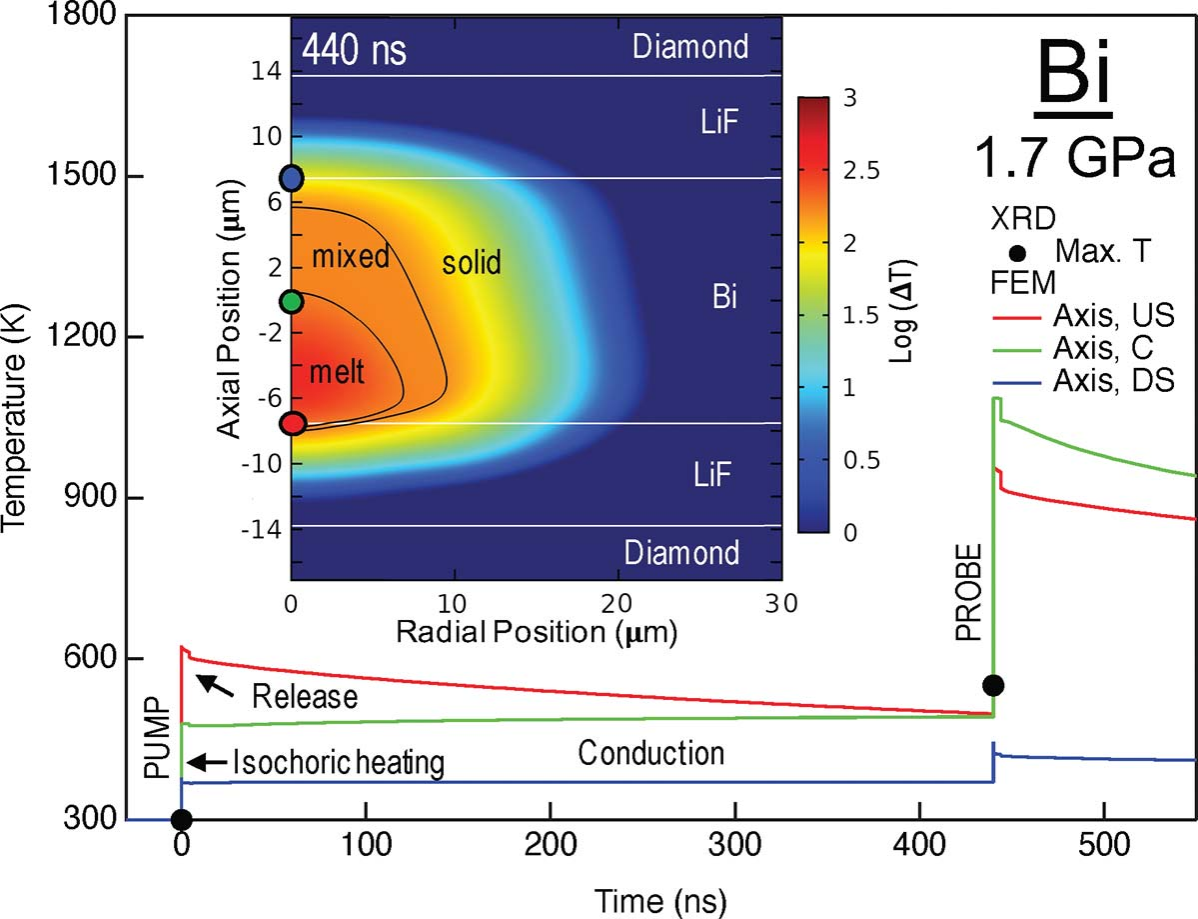
Temperature history in the Bi sample at 1.7 GPa and 3% transmission based on XRD and finite element modeling. The maximum temperature determined from the thermal expansion model based on diffraction is indicated by black dots. Based on the energies entering the beamline during the shots, 658 (43) µJ at XGM_SA2, we computed the local equilibrium temperature due to isochoric heating, hydrodynamic release and conduction (Meza-Galvez *et al.*, 2020 ▸), including latent heat of melting through the effective heat capacity model and radiative cooling (Gomez-Perez *et al.*, 2017 ▸). Temperature histories at three positions along the axis are shown as colored lines. The inset shows the axisymmetric temperature distribution in the sample area at the time of the probe. The black lines are isotherms indicating the edge of the mixed-phase region in Bi and the colored dots indicate the positions of the corresponding temperature histories. The model uses thermal parameters [see Meza-Galvez *et al.* (2020 ▸) for notation] appropriate for hot compressed Bi [ρ = 10.22 g cm^−3^, *C_V_* = 120 J kg^−1^ K^−1^, *C_P_* = 139 J kg^−1^ K^−1^, *k* = 8 W m^−1^ K^−1^, γ_0_ = 1.25 (Gorman *et al.*, 2015 ▸), β = 4 × 10^−5^ K^−1^, melting temperature of 470 K (Ono, 2018 ▸), latent heat of 52 kJ kg^−1^ and radiative emissivity of 0.33]. Values for the LiF PTM, diamond and gasket are taken from Meza-Galvez *et al.* (2020 ▸). X-ray absorption (and attenuation) factors at 17.8 keV are 119 048 m^−1^, 275 m^−1^ and 161 m^−1^ for Bi, LiF and diamond, respectively. Diamond thicknesses were 1.7 mm, the cavity thickness determined from white-light interference was 27.5 µm and the Bi layer thickness measured by X-ray absorbance was 15 µm.

Calculation of the Bi sample temperature based on this incident energy (Fig. 14 ▸) shows that, through isochoric heating and relaxation between pump and probe pulses, the bulk sample temperature remains close to the melting point, due to latent heat effects and a large mixed-phase region in the sample. This explains both the XRD observations of crystalline Bi close to melting and the presence of a strong diffuse scattering signal from the liquid. The sample remains warm (>10 K heating) for ∼30 µs after the exposures, reiterating the importance of considering heat deposition, cumulative heating and cooling dynamics during high-repetition-rate pulse trains, particularly for high-*Z* samples.

### 4.5. Diamond stability   

One of the critical questions discussed by Liermann *et al.* (2016 ▸) concerned the stability of the diamond anvils in the X-ray beam of the HED instrument at EuXFEL. Exposing different types of diamond anvils during the 1st UCAC experiment has provided some information about the stability of the diamond anvils in a 17.8 keV X-ray beam as a function of fluence, sample, the PTM, and the stress state (pressure) of the diamond anvil. Table 5 ▸ gives an example of some of the types of diamond anvils and the conditions to which the DACs were exposed during the 1st UCAC experiment. In general, this table indicates that the diamond anvils are remarkably robust and can survive 2.2 MHz X-ray beam exposure at 17.8 keV and maximum fluence when the beam is relatively large (10–20 µm FWHM). This is even true when supporting pressures above 100 GPa and exposed to many multi-pulse trains at 10 Hz over many seconds. In the few cases where diamond damage was observed it originated from the alignment of the X-ray beam in correspondence with damage imprints on the gasket materials. In such cases, the heated ‘sample’, *i.e.* gasket, was in direct contact with the diamonds without any insulating effect of intermediate PTM. Thus, sufficient insulation of the sample through low-*Z* PTMs is essential to avoid damaging the diamond anvils. However, in one case the exposure of a sample consisting of a low-*Z* olivine silicate led, after very long exposure, to the cracking of the diamond.

## 5. Outlook   

While a large part of the DAC setup in IC2, such as the sample stack, the VAREX detectors *etc.*, has been commissioned and successfully used during the 1st UCAC experiment (proposal No. 002292), there are still some components that require further improvement and installation. One of the major challenges during the 1st UCAC experiment was the determination of the actual X-ray beam energy and intensity (energy) that was incident on the sample via the HIREX-II and calibrated DIMs directly in front of and behind the DAC, respectively. Future calibration of these DIMs will help to estimate more accurately the temperatures that might have been reached during X-ray heating and compare them with the SOP data, thereby gaining a much better understanding of the overall process of X-ray heating in a DAC. The most important addition to the setup, however, will be the 1 Mpixel AGIPD that will enable the collection of a diffraction pattern resulting from each X-ray pulse, rather than the summed and overlapped diffraction patterns obtained in the 1st UCAC experiment. This will not only help to clarify the X-ray heating effects (using precise peak-position measurements to estimate the temperature of the sample) but also enable the use of pulsed optical laser heating, as well as dynamic compression experiments in a dDAC at intermediate strain rates of up to 10^3^ or 10^4^ s^−1^. Furthermore, with improved pointing stability, future experiments will benefit from the implementation of a pinhole immediately in front of the sample to clean up the tails of the focused X-ray beam.

As the operation of the EuXFEL matures, higher X-ray energies, up to 25 keV, will become available, providing greater access to *Q* space. The lower fluences expected at higher energies make this option particularly attractive for pulse laser heating and dDAC X-ray diffraction experiments of high-*Z* compounds, where X-ray heating is not desired and, due to the increased energy, will be reduced. Finally, heating samples with the X-ray beam can potentially open up a novel way of exploring the high-pressure and high-temperature properties of low-*Z* or otherwise difficult to heat materials (*e.g.* infrared laser reflecting metals such as Au) in the very near future.

Besides XRD experiments in IC2 there are ongoing efforts to perform X-ray spectroscopy experiments (*e.g.* emission spectroscopy) in IC1 in a DAC and, in the mid-term, X-ray PCI experiments combined with XRD and spectroscopy in both interaction chambers.

## 6. Conclusions   

During the first 1st UCAC experiment (proposal No. 002292) a cross section of the international static high-pressure community came together to commission successfully the DAC precision diffraction setup within IC2 of the HED instrument at the European XFEL, using VAREX XRD 4343CT flat-panel detectors. Experiments using X-ray pulses at 17.8 keV enabled collection of XRD images in 20 fs from a sample in a DAC, while two serial pump–probe exposures utilizing the intrinsic bunch structure of the European XFEL demonstrated the effects of heating by the first pulse, as revealed in Bi placed under pressure in a DAC. The experiments demonstrate that diamond anvils and confined samples are robust under hard XFEL irradiation, enabling serial interrogation and excitation.

## Figures and Tables

**Table 1:** Possible repetition rates (fraction of 4.5 MHz), number of pulses (detector limited) and the resulting length of the pulse train that will limit the duration of the experiment

In-train repetition rate (MHz)	Number of pulses (detector limited)	Maximum length of the pulse train (µs)
0.563	338	600
0.75	350	467
1.1	350	318
2.2	350	160
4.5	350	77.8

**Table 2:** Translations and their specifications used for the sample stack inside IC2

Name	Part No. / manufacturer	Travel/reproducibility/accuracy (mm/µm/µm)	Load capacity (N)
CenX	510210-X1.HV / Huber	12/0.1/0.1	500
CenZ	510210-X1.HV / Huber	12/0.1/0.1	500
SamY	NPE-200 / PI	13/0.04	300
Omega	409-X1W1.HV / Huber	360/0.00145/0.00145†	500
SamX	510130-050X1.HV / Huber	50/1/1	500
SamZ	510130-450X1.HV / Huber	450/1/1	500

† Units are in °/mrad/mrad.

**Table 3:** Maximum coverage that can be achieved in reciprocal space with the final AGIPD detector setup and coverage reached during the 1st UCAC experiment using the VAREX XRD 4343CT flat-panel detectors

Energy (keV)	SDD (mm)	2θ coverage (°)	*Q* (Å^−1^)
25	150	45	9.695
17.818	257.3	45	6.910
17.818	257.3	35	5.430

**Table 4:** Detailed specification of incident beam and Bi-I unit cell parameters determined from the integrated diffraction patterns Cell parameter refinements were performed using *REFINE* (Bartelmehs & Downs, 1996 ▸) after determining the diffraction peak position using the program *PeakFit*. Volume is given per unit cell of Bi-I. The refinement of the cell parameters for the cold and hot states of Bi-I in diffraction patterns R0427 and R0425 are based on the three strongest diffraction peaks, resulting in an underestimation of the errors of the cell parameters, pressure and temperature. For the refinements of R0431 and R0442 all reflections shown in Fig. 13 ▸(*a*) are used, resulting in more realistic error estimation.

					Cell parameters					
Run No.	Pulse	Transmission (%)	Diode PD2 (ADUs)	HED-XGM (µJ)	*a* (Å)	*c* (Å)	*V* (Å^3^)	Diffraction *P* (GPa)	Diffraction *T* (K)	Liquid peak (Å^−1^)
R0431	1	3%	2268	700	4.509 (1)	11.605 (3)	204.31 (9)	1.49 (1)		
R0442	1	3%	2863	650	4.511 (1)	11.624 (5)	204.9 (1)	1.38 (2)		
R0424	1	1%	311	690	4.506 (2)	11.574 (5)	203.5 (1)	1.66 (2)		
	2		1270							None
R0427	1	3%	739	700	4.5007 (9)	11.593 (7)	203.36 (7)	1.68 (1)		
	2		831		4.5068 (1)	11.6750 (9)	205.36 (1)		552 (6) K†	Weak
R0425	1	5%	2787	580	4.5043 (8)	11.5924 (4)	203.684 (5)	1.619 (1)		
	2		3884		4.5078 (2)	11.689 (1)	205.67 (1)		551 (2) K†	13.35 (1)
R0428	1	10%	8517	690	4.5048 (7)	11.575 (3)	203.41 (5)	1.67 (1)		
	2		6425						All melt	13.35 (3)

† Temperature is estimated from the thermal expansion of Bi-I phase collected at 5 K and ambient pressure by Fischer *et al.* (1978 ▸).

**Table 5:** Examples of the types of diamond anvils, samples and PTMs, the maximum exposure conditions, and the nature of any damage to the diamonds

DAC	Diamond type†	Sample / PTM	*P*_max_ (GPa)	Fluence_max_ (µJ)	No. of pulses_max_	Damage type
S2	Ia, BA70, 0.2 mm	10 µm Cu foil / MgO	55.5	158	20	None
HIBEF32	IIa, ST, 0.3 mm	10 µm Zr foil / NaCl	9	196	20	None
SBU2	Ia, ST, 0.3 mm	Olivine powder (Fo_55_Fa_45_)	30	203	10	None
SB001	Ia, MBC, 0.5 mm	4 µm Fe foil / N_2_	5	205	20	None
VP002	Ia, BA70, ST, 0.25 mm	Au (black) powder / H_2_O	40	130	20	Damage during alignment (upstream side)
HIBEF03	IIa, BA70, ST, 0.2 mm	2 µm Au foil / H_2_O	30	152	20	None
HIBEF04	IIa, BA70, ST, 0.25 mm	12 µm Mo foil / LiF	29	204	20	Damage during alignment (upstream side)
HIBEF05	IIa, BA70, ST, 0.15/0.3 mm	2 µm Au foil / Quartz	113	121	20	None
HIBEF30	IIa, BA70, ST, 0.3 mm	Olivine powder (Fo_90_Fa_10_) / Ne	35	214	20 × 110 = 2200 (20 @ 10 Hz)	Diamond cracked

† BA70 = Böhler Almax design with 70° opening. ST = standard design. MBC = modified brilliant cut.
